# Sexual transactions between long distance truck drivers and female sex workers in South Africa

**DOI:** 10.1080/16549716.2017.1346164

**Published:** 2017-08-02

**Authors:** Nosipho Faith Makhakhe, Tim Lane, James McIntyre, Helen Struthers

**Affiliations:** ^a^ Department of Psychology, School of Applied Human Sciences, University of KwaZulu-Natal, Durban, South Africa; ^b^ Department of Medicine, Center for AIDS Prevention Studies, San Francisco, CA, USA; ^c^ Anova Health Institute, Johannesburg, South Africa; ^d^ School of Public Health & Family Medicine, University of Cape Town, Cape Town, South Africa; ^e^ Division of Infectious Diseases & HIV Medicine, Department of Medicine, University of Cape Town, Cape Town, South Africa

**Keywords:** Female sex workers, long-distance truck drivers, HIV, antiretroviral treatment, South Africa

## Abstract

**Background**: Female sex workers (FSWs) and long distance truck drivers (LDTDs) are considered key populations at high risk for HIV transmission due to high prevalence. The intersection of these mobile populations presents unique challenges in the fight against HIV and the movement towards reducing new infections.

**Objective**: The purpose of this study was to explore the nature of sex trade along a particular transport route. Sexual transactions and the vulnerabilities that exist between these two groups with regards to HIV/AIDS are described, with the purpose of furthering the agenda for targeted interventions.

**Methods**: Qualitative in-depth interviews were conducted with 14 participants, seven FSWs and seven LDTDs. We recruited FSWs through snowballing, and LDTDs through intercepts at truck stops. Semi-structured interview guides were used for data collection, and thematic analysis was conducted.

**Results**: The sex trade in this study is characterized by competition, fuelled by money-driven and age-disparate rivalry. Despite widespread HIV knowledge, FSWs contend with persistent challenges regarding condom use negotiation, induced by more money in the exchange for unsafe sex. Despite the placement of wellness centres in truck stops along the highway, LDTDs face stigma related challenges with regards to testing for HIV and personal acknowledgement of their involvement in the sex trade.

**Conclusion**: The nature of the sex trade along the highway continues to be risky despite the availability of HIV testing and antiretroviral treatment (ART). The sex trade is perceived to be increasing along trucking routes, in spite of measures instituted to limit access to FSWs. FSWs struggle to cope with the pressure of unprotected sex because of the need to generate more income, as well as avoid incidents of violence and threats. Interventions along transport routes need to be inclusive of FSWs who could play a vital role in stigma reduction amongst LDTDs through peer education.

## Background

In recent years, countries in Eastern and Southern Africa have seen a significant decline in the number of new HIV infections, from 1.7 million in 2001 to 1.2 million in 2011.[1] Countries such as Swaziland and South Africa have shown rates of decline in new infections ranging between 25 and 49% in the adult population in 2001–2011.[1] These remarkable declining trends can be attributed to protective factors such as increased condom use, increased access to antiretroviral treatment (ART), and progress in preventing mother to child transmission.[1] However, key and vulnerable populations, such as female sex workers (FSWs), men who have sex with men (MSM), persons who inject drugs (PWID) and long-distance truck drivers (LDTDs), are regarded as the residual drivers of HIV transmission.[2,3] Additional efforts are needed to address the susceptibility of these key and vulnerable populations to new HIV infections and transmissions.[3,4]

In general, key populations are poorly represented in national HIV surveillance studies and health care interventions because they are perceived as difficult to access.[3,5,6] FSWs, by the nature of their work, are exposed to stigmatization, discrimination, and vulnerability to arrest, which inhibit them from seeking health care.[5–9] As a consequence of high numbers of their multiple sexual partners, high frequency of sexual exposure, and low levels of condom use, FSWs are at high risk of contracting HIV and other sexually transmitted infections.[10–12] A recent study conducted along the N3 highway between Kwa-Zulu Natal and Free State in South Africa reported 90.6% HIV prevalence among FSWs.[13] Furthermore, FSWs are exposed to gender-based violence and physical abuse, which places them at even greater risk of HIV infection.[5,9,14–16] Therefore, it is no wonder that FSWs are 13.5 times more likely to be living with HIV than other women, and are designated as a key population in relation to the HIV epidemic.[1,5,17–20] Yet, the industry of sex work continues to thrive despite the high prevalence of HIV among FSWs, with the continued risk of transmission to their clients who include other vulnerable groups, such as LDTDs.[2,12]

LDTDs have long been understood to be highly vulnerable to HIV infection and onward transmission of HIV along major trucking routes.[21] However, the susceptibility of LDTDs to HIV acquisition is increasingly being acknowledged, with a prevalence of 56%,[22,23] resulting in their designation as a vulnerable population in need of targeted interventions.[2,23] Most studies have investigated either FSWs [6] or LDTDs independently,[23] with little acknowledgement of the intersection between these two groups.[12] The interaction between FSWs and LDTDs is often conceptualized geographically as a high HIV transmission area.[2,3,21,23] However, we know very little about the nature of sexual transactions between FSWs and LDTDs, especially in this era of widely available HIV testing and the large-scale access to ART. Studies looking at the interaction between FSWs and LDTDs in South Africa were conducted long before the advent of ART,[21,22] indicating the need for further research.

More recent studies suggest that there are various structural factors that contribute to high HIV rates in FSWs,[6,24] and LDTDs.[2,23] Poverty is a driving force of HIV transmission in women in sub-Saharan Africa; growing national-level income inequalities are associated with higher HIV prevalence.[25,26] Women who enter sex work along transport routes are characterized by poverty and unemployment, turning to sex work as a means for survival.[13] Likewise, the nature of their employment, being on the road for extended periods of time away from intimate partners, renders LDTDs more likely to solicit FSWs along transport routes.[2,23] Therefore, sexual dynamics between these two groups need to be further understood. This study was conducted mainly to explore the nature of the sex trade along a particular highway. We also explored sexual transactions and vulnerabilities manifesting at the nexus between FSWs and LDTDs, with the purpose of furthering the agenda for targeted and tailored health promotion and interventions.

## Methods

### Study setting

This study was conducted along the N3, a major highway between Johannesburg and Durban. This route consists of numerous truck stops and gas stations that provide lodging and refreshment facilities for LDTDs transporting goods between the two major cities. The sex trade in this instance occurs in the vicinity of gas stations situated on either side of the highway, with designated parking areas for LDTDs to park their trucks.

### Research design

Qualitative methods were adopted to explore and understand the nuances of social life as well as the subjective meaning that people attach to their actions.[27] We therefore used the constructivist paradigm, which enables inquirers to understand the world in which individuals live and work.[27] Individual experiences create meaning, which informs their beliefs and understanding of the world, and it is the understanding of the sex trade from the perspectives of FSWs and LDTDs that the current study sought to capture.[27]

### Sample and sampling strategy

The sample consisted of 14 key informants, seven males between the ages of 18 and 53 years and seven females between the ages of 21 and 40 years. Six males were long distance truck drivers and one male was a bartender of an establishment frequented by both FSWs and LDTDs. All the females were FSWs. Convenience sampling was adopted to recruit LDTDs on the basis of their willingness to participate; LDTDs were intercepted at various truck stops along the N3 highway. Snowballing was used in the recruitment of FSWs at an identified hotspot along the highway. The researchers approached one FSW who agreed to participate and was willing to direct researchers to other FSWs. Participants were selected based on the following inclusion criteria: aged 18 years and above, self-identified as a FSW, LDTD, or as someone familiar with FSW and LDTD populations in the area; agreed to participate in the study; and willing to answer questions related to their experiences.

### Data collection

The data were collected between October and November 2013. Two female researchers trained in qualitative interviewing conducted face to face interviews using a topic guide to collect the data. The interview guide covered topics pertaining to sexual practices, health behaviours, access to health care, HIV knowledge and knowledge of HIV status, and the frequency of truck drives on the road. This enabled participants to speak about their experiences and follow up probe questions were used to further explore participants’ viewpoints. Interviews were conducted in the participant’s preferred language of communication, which was either IsiZulu or English, and lasted 30–45 minutes. Data saturation was reached when the researchers noticed consistencies in the data, when data within each theme from various participants became repetitive.

### Data analysis

Audiotaped data were transcribed and translated verbatim from isiZulu to English. Data were analysed using a thematic approach that involved sorting and coding the data into themes and categories by identifying and analysing repeating patterns in the data.[28] The first author coded the data and after extensive consideration of the coded transcripts consensus was reached among the four authors in terms of the emerging themes from the data. The analysis followed the six steps as outlined by Braun and Clarke,[28] to translate and transcribe the data verbatim, familiarizing oneself with the data by reading and re-reading the data so as to generate codes, generate themes from the codes, define and refine the identified themes, and employing the identified themes in the final presentation of study findings.

### Ethical considerations

Ethical permission was obtained from the Ethical Review Board of Cape Town, Cape Town, South Africa [UCT HREC Reference # 091/2013] and was determined by the USA Centres for Disease Control Division of Global HIV/AIDS Program as non-engaged research. Written informed consents were obtained from all participants. Identifiers such as names of truck stops and area names of sex work hotspots were removed from the data presented in this study in order to ensure confidentiality.

## Results

Using thematic analysis, three broad themes were identified: the nature of the sex trade, sexual encounters and preferences, and difficulties of access to health care.

### The nature of the sex trade

#### Female sex workers

The FSWs in this study were women aged 21–40 years. They described the sex trade along the highway as being characterized by territorial boundaries and market price regulation. These women were organized such that younger women below the age of 30 years worked at one gas station for trucks traveling northwards, and older women aged 30 years and above worked on the opposite side of the highway for trucks travelling southwards.

'Well to start with, one round is R50 (‘R’ refers to the South African Rand, which is the local currency). When a person says that well I do not use condoms, it then depends on you as a woman on what you will choose to do. I will speak of working arrangement here. Us grown ladies work on this side of the garage, and the younger ladies work on the other side. When a young lady is new and comes unknowingly to this side, we immediately tell her to move to the other garage. Reason being, when we older women, between the ages of 32-38 and 40s, work with the younger women, then it becomes difficult for us to make money. Older men love younger women. That’s why we decided to work on separate sides.' (FSW 1, aged 40 years)

Older women found their work less productive when they shared the same spot as younger women. These boundaries were respected and honoured by both groups, and any violation was met with threats and incidents of physical violence and assault, as was demonstrated by FSW 6, aged 21 years; ‘The old ones want to beat us up, and they get jealous because we are still young and we get more clients. So it’s best we separate’. Furthermore, the price of sex was fixed at R50 per session and R250 for the whole night for both age groups.

FSWs mentioned that they were threatened with violence from the hands of clients who refused to pay after a service has been rendered.

'Yes, at times the men complain and say that they do not have enough money, and want us to drop the price to R40. Some sleep with you, and they do not want to pay. If you insist they take out a gun or knife and threaten to kill you.' (FSW 2, aged 38 years)

#### Long-distance truck drivers

LDTDs in this study, aged between 30 and 53 years, were not forthcoming about paying for sex, because they felt that there was a general perception that because they were truck drivers they all solicited the services of FSWs. Thus when questioned about picking up FSWs they refrained from speaking personally, but narrated experiences of other drivers who they had seen pick up women. They did, however, indicate that the buying of sex occurs frequently. Most importantly, some believed that the sex trade between FSWs and LDTDs could not be curtailed, as LDTD 3, aged 53 years, claimed with conviction that

'[Truck drivers] will not stop doing it, it still happens. It’s something that drivers are used to doing, they will not stop.' (LDTD 3, aged 53 years)

Another LDTD expressed concern that the sex trade remains rampant despite the scourge of HIV/AIDS, but safer sex practices were needed.

'In my opinion the demand for sex workers among truck drivers is growing every day. I don’t know how these ladies feel making money through sex work. I think sex workers should be registered and sex work be monitored so that it is made safer, because it is on the rise and it is continuing. So the only way is to make sure that sex workers and their clients are encouraged to have safe sex. Right now sex workers have no track record and are not monitored so it makes it dangerous.' (LDTD 7, aged 30 years)

Although LDTD 7 above calls for recognition of sex work and safer sex practices, he also expressed contempt for sex work as well as sentiments of moral judgment and condemnation.

In an effort to further distance themselves from association with FSWs, the LDTDs in this study were adamant that due to strict surveillance policies through the use of cameras inserted inside the trucks, it is not possible for them to pick up FSWs or any other passengers on the road.

'What I can tell you is that even when we are seated now talking there are people who see us through cameras, so the trucks also have cameras. Even when you stop to take a piss they will ask you what you are doing. Because we transport petrol security is very tight.' (LDTD 2 aged 52)

However, FSWs claimed that cameras did not prevent drivers from picking up women, as was revealed by FSW 4, aged 23 years, ‘What he does is he will block the camera lens using tissue. And then we come in’ (FSW 4), and FSW5, aged 22 years, ‘The guys that drive trucks with cameras buy sex at night. They close the camera with curtains’. Furthermore, security measures at designated truck stops prevented women from walking in, lest they be suspected of sex work. However, FSWs revealed that the pick-up happens before the LDTD gets to the truck stop, and that way no one questioned them when they drove in with a woman, who could be their partner or wife.

### Sexual encounters

#### Female sex workers

According to FSWs in our sample, LDTDs have various sexual preferences including dry vaginal sex, vaginal sex, oral sex and anal sex. FSWs were more averse to providing oral sex than they were to anal or vaginal sex, because they deemed oral sex to be more risky, as was expressed by FSW2, aged 38 years, ‘Some say look the other way [anal], others want to be sucked. I say no to sucking because there are all sorts of diseases out there’ (FSW 2), and FSW 4 said, ‘I will suck provided that the guy wears a condom’ (FSW 4). FSWs were preoccupied with navigating how to provide what the client wants.

'Different clients prefer different styles, it’s up to you if you will offer them what they want. If a client has a certain style that they want and you are not willing to do [it] then he moves on to the next woman.' (FSW 1)

FSWs were less knowledgeable about safer anal sex practices, such as using water-based lubricants with condoms. Instead, they used oil-based substances such as dawn (body lotion) to provide moisture, unaware that these contribute to condom breakage. Furthermore, all but one of the FSWs said that they came across clients who refuse to use condoms or offer more money for sex without a condom.

'You will first look at him and if you see that he seems healthy, even though you cannot know for sure because you do not know his status, you will say yes to sex without a condom, and he will give you R100 [doubled price]. But if you feel uncomfortable to do so, you say no. It depends on the sort of person you are.'(FSW 1)

FSWs appraised the client physically prior to making a decision, knowing fully well that health status could not be determined in such a way. They suggested that this process of decision-making was dependent on the character and preferences of individual FSWs. Some FSWs refuse sex without a condom, even for over and above the fixed price, due to a high perception of risk and fear of contracting disease, as shown below.

'One client once said he doesn’t use condoms. Obviously he’s got a disease. Some sleep with clients without condoms. I would never do that. Yes the condom can burst while we [are] doing it, but I would never start off without a condom. R500 is too small compared to my life.' (FSW 2)

The responsibility to carry condoms rested largely with the FSWs. Condoms were largely obtained for free from local clinics, but they reported that nurses would not allow FSWs to take the number of condoms they actually needed and they were stigmatized. The majority of FSWs mentioned that they had to ensure that condoms were available, but that some clients do not want to use free condoms. Some FSWs mentioned that when they run out of free condoms, they are forced to purchase them at the gas station, and they are expensive.

#### Long-distance truck drivers

LDTDs were not open to discussing condom use in personal sexual encounters, but mentioned that they were able to access free condoms at the mobile clinics inside the truck stops. One LDTD was emotive in making a statement about the lack of condom use, but in reference to other LDTDs, saying:

'I am going to be brutally honest with you, you might not like what I have to say. I have worked with two guys who tested positive, but they do not use protection. You can hear from the way they speak, where one will say ‘agh! That guy is dying with me’. We have all slept with the same woman. So, some are reckless and careless. I know those guys they are still living today. It’s a long way to go before we can all be responsible.' (LDTD 7)

The same LDTD later mentioned that he had not taken condoms from the dispenser in a long time, because he wanted to restrain himself from picking up women.

'Yes I buy them [condoms] but keep them at home in that way I can restrain myself and not do what I shouldn’t do.' (LDTD 7)

The analysis of responses from LDTDs shows that there was an inherent feeling among LDTDs that admitting to being in possession of condoms would automatically mean that they were soliciting FSWs, or that they were somehow unable to control themselves.

### Access to healthcare

#### Female sex workers

FSWs accessed healthcare at their local clinics where they reside, although they work and move between truck stops, which have wellness centres. These wellness centres cater specifically for LDTDs and provide basic HIV counselling and testing services as well as routine vital checks. FSWs reported finding it difficult to gain access to these wellness centres because they are not allowed to enter truck stops.

Several participants noted that FSWs had in the past used the wellness centres as a way of gaining entry to the truck stops and thereafter to solicit clients. This practice made security at these truck stops wary of letting them gain access to the wellness centres. Gaining access to these wellness centres could help FSWs replenish their supply of free condoms without having to purchase them. These facilities could be convenient for HIV testing as well as treating other STIs.

Nevertheless, FSWs in our sample mentioned that they regularly tested for HIV. They spoke of HIV freely, treating it as a chronic illness. The majority of FSWs knew their HIV status, going as far as taking their ART in front of one another devoid of fear or stigma.

'Well most women know their status. But I don’t think that they would have a problem with testing. I always see large numbers of women taking treatment at different set times.' (FSW 1)

FSWs were conscious of HIV because the threat of acquiring infection was a real danger that they contended with on a daily basis, due to risky sexual practices such as unprotected sex and mishaps such as condom breakage.

#### Long-distance truck drivers

Several LDTDs in our sample mentioned that HIV testing was a great source of discomfort and if given the choice to test most LDTDs would refuse.

'A lot of people talk about it but few have actually gone to test. I think maybe about 20% of people have actually tested, maybe. I mean the majority of people I know, even those who are not drivers, speak about HIV but are never forthcoming whether they have actually gone to test.' (LDTD 7)

Some trucking companies provided testing services but, unlike FSWs, LDTDs did not talk openly about HIV or testing. However, when challenged to test, LDTDs may be willing to face their fears in order to prove their prowess.

'I am not scared, I was about to go to the wellness centre to do it. I have done it before and I am still going to do it again. I am not scared. But it is a subject that people are still sceptical about. In my previous company they used to provide HIV testing. HIV testing is stigmatized.' (LDTD 7)

The biggest barrier for LDTDs, in spite of availability of services, is HIV-related stigma. Unlike FSWs, who were free to talk about and test for HIV but had limited health services, experiences of LDTDs suggest availability of services, but poor uptake due to HIV stigma.

## Discussion

This study found that the sex trade along the highway connecting Durban and Johannesburg in South Africa was highly territorial, competitive and sometimes violent. In the backdrop of rivalry, FSWs continued to engage in risky sexual behaviour so as to win over or please their clients. Most were willing to serve clients who refused to use condoms, and they accepted more money for riskier sex in spite of their extensive knowledge of the increased risk of transmission. Some FSWs gauged risk by inspecting the client’s physical appearance. LDTDs were relatively less knowledgeable about the risks of HIV transmission, shy to discuss involvement in the sex trade, and showed a fear of associated stigma for both HIV and FSWs. While HIV testing is widely available for LDTDs in the wellness centres within the truck stops, most are still reluctant to test and hesitant to collect condoms. FSWs are not allowed access to these wellness centres. Furthermore, security measures have been put in place, which could dissuade LDTDs from picking up FSWs. However, LDTDs have also found ways of evading current security measures, as witnessed by FSWs.

Studies have shown that LDTDs and FSWs intersect along transport routes to exchange sex for money.[21,22,29,30] In this study, the sex trade along the highway was characterized by age-disparate rivalry and territorial boundaries. Territorial segregation was established to limit the intensity of competition. LDTDs observed the sex trade as a growing industry with no immediate end in sight. As mentioned by FSWs, security measures have not discouraged LDTDs from soliciting their services, and LDTDs have found ways to bypass these systems. While they engage in the highway sex trade, LDTDs in our sample deflected the buying of sex onto other fellow LDTDs; this could be interpreted as their way of avoiding social undesirability associated with picking up FSWs. A study conducted over 20 years ago by Abdool Karim et al. [22] showed that LDTDs were reluctant to speak about their personal lives or issues of HIV/AIDS for fear of prosecution or loss of employment. Evidently years later truck drivers are still showing a similar reluctance to disclose engagement with FSWs along trucking routes.

This study also found that FSWs are knowledgeable about HIV and safe sex practices, but did not always prioritize health over other concerns when faced with challenging situations, such as clients willing to pay more for sex without a condom. Abdool Karim et al. [22] recorded a similar finding among sex workers trading along the highway between Durban and Johannesburg. This is a demonstration of the gap in terms of knowledge and behavioural change among FSWs, which current HIV interventions need to bridge. Previous studies have argued that failure by FSWs to negotiate condom use with clients is a result of powerlessness; these studies further suggest that this powerlessness is mediated by both economic and physical safety concerns.[31–35] By insisting on safe sex, FSWs risked physical and sexual abuse from clients and loss of possible income, a pattern well demonstrated in the literature.[7,22,36–38] The prospect of receiving more money and avoiding violence creates pressure that incentivizes FSWs engagement in risky sex.[11,19,21,36,38,39] A study by Malta et al. [40], conducted in Brazil showed that in moments of pressure and uncertainty, FSWs relied on physical assessments to determine HIV serostatus, despite high levels of HIV knowledge. Similarly the FSWs in our sample described similar behaviour as seen among their peers despite the knowledge of risks associated. The highly competitive nature of the sex trade along this particular transport route, coupled with the need to make financial ends meet, likely hinders FSWs from uniting and instituting their own protective laws that govern the trade.[22,35]

In this study, FSWs felt that the responsibility to carry condoms rested largely with them. Some clients did not carry condoms, and at times threatened violence if FSWs insisted on condom use, as found in other studies.[5–7,22,33,39,40] LDTDs are known to have high HIV rates, largely due to irregular condom use with FSWs.[21,22,40] As seen in other studies some LDTDs are more likely to report condom use with a one-night stand as opposed to regular FSWs with whom they have an attachment.[36,40] The necessity to practice safe sex is in time substituted by feelings of trust, which conjures the need for closeness, resulting in unprotected sex.[30,41] In this study some FSWs mentioned that they come across clients who refused to utilize free *Choice* condoms provided by the South African government at public health facilities, insisting on commercialized brands costly for FSWs to buy.[39] A study by Baker et al. [42] explored custom-fitted male condoms as a sexual health intervention in Cape Town, and outlined concerns expressed by heterosexual males with regard to use of free government (*Choice*) condoms, possibly relevant to truck drivers in our study. Data showed that 67% of heterosexual males complained largely of condom fit, function, breakage as well as slippage.[42] FSWs in the same study confirmed that males preferred coloured, textured as well as flavoured condoms as opposed to the beige and bland government condoms.[42] These findings by Baker et al. [42] could provide explanation for some of the difficulties experienced by the FSWs in attempting to insist that clients use condoms. The government is however currently involved in the rebranding of *Choice* condoms to improve user desirability.

The well-known stigmatization associated with a positive HIV status [6,19] was not reflected among the FSWs in our sample, of which we speculate the majority to be HIV positive, as demonstrated by the finding that they all take their ART in front of each other. This marginalized group has fostered an environment of open dialogue on issues of HIV. This observed openness could be nurtured and encouraged, so that FSWs along transport routes can convey HIV awareness even to their clients. A study by Morris et al. [43] mentions that mobile populations should not only be viewed as bridge populations to the spread of HIV, but should also be seen as potentially effective in spreading messages on condom use and HIV education. Even though LDTDs have access to convenient health care in the form of wellness centres situated in various trucks stops along the highway, this access has not made HIV testing any easier.[23] LDTDs experience feelings of fear and shame, which hinder them from seeking the help they need. Thus they may unknowingly continue to spread HIV to their partners who are not in the sex trade.

## Study limitations

The findings from this qualitative study draw on a small sample to provide an in-depth understanding of the data and are not intended to be statistically representative. The small sample of LDTDs was interviewed by a female interviewer, which could have hindered LDTDs from sharing personal experiences pertaining to FSW engagement. Future studies with larger sample sizes could address minority views identified in this study, such as willingness of LDTDs to test for HIV, disclose engagement of FSWs and admit to use of illicit substances. Having a LDTD peer interviewer, a male researcher or a FSW interviewer could have yielded different results, as seen in other studies.[4,22] The FSWs in this study were recruited from the same hotspot and so the study could have missed dynamics of the sex trade taking place in other parts of the N3 highway and other transport routes in South Africa. Thus results from this study are unique to this setting and cannot be generalized to other settings where FSWs engage with LDTDs.

## Conclusion

The nature of the sex trade between FSWs and LDTDs along the highway continues to be risky for HIV acquisition despite the availability of HIV testing and ART. The sex trade is perceived to be increasing, and remains highly competitive and volatile, although trucking companies and truck stops have instituted measures to limit LDTDs’ access to FSWs. FSWs struggle to cope with the pressure of unprotected sex because of the need to generate more income, as well as avoid incidents of violence and threats. It is however encouraging to note that FSWs openly test and take HIV treatment in front of their peers. While truck drivers have widely available services, there is continued need for education, awareness of and testing for HIV. They are a bridge population and need to protect their regular partners who are at risk of sexually transmitted infections. Interventions along transport routes need to be inclusive of FSWs who could play a vital role in stigma reduction amongst LDTDs through peer education. Policies at truck stops should be reviewed to allow FSWs access to HIV testing at wellness centres and be encouraged to bring their partners who are LDTDs to test. FSWs organizations are not active along transport routes but should support these isolated FSWs so that they can benefit from the social as well as the legal support. Further research is needed in understanding the reluctance of clients to use government supplied condoms, as well as how PrEP (pre-exposure prophylaxis) will change the dynamics of the sex trade.

## Paper context

Female sex workers (FSWs) and long distance truck drivers (LDTDs) are considered key and vulnerable populations with regards to HIV. This study indicates that even though FSWs trading along the N3 highway have limited access to tailored HIV care services, they are knowledgeable about HIV and confess to taking antiretroviral treatment. However this knowledge does not necessarily translate into safe sex behaviour. Conversely, LDTDs have access to HIV care services at truck stops, but some are hesitant to test for HIV due to stigma. Sexual transactions between these two groups continue to occur despite the known associated risks of HIV transmission.
